# Step-Determined Physical Activity among Individuals with Chronic Conditions: The 2007-2019 National Health and Nutrition Survey of Japan

**DOI:** 10.31662/jmaj.2025-0122

**Published:** 2025-08-22

**Authors:** Noritoshi Fukushima, Shiho Amagasa, Misaki Takahashi, Hiroyuki Kikuchi, Rei Ono, Shigeru Inoue

**Affiliations:** 1 Department of Preventive Medicine and Public Health, Tokyo Medical University, Tokyo, Japan; 2 Graduate School of Public Health, Teikyo University, Tokyo, Japan; 3 National Institutes of Biomedical Innovation, Health and Nutrition National Institute of Health and Nutrition Center for Physical Activity Research, Osaka, Japan

**Keywords:** clinical guidelines, comorbidity, exercise, non-communicable diseases

## Abstract

**Introduction:**

Clinical guidelines recommend exercise therapy to modify an unhealthy lifestyle among individuals with chronic conditions (CCs), including hypertension, diabetes, and dyslipidemia. However, it remains unclear whether individuals with CCs engage in higher physical activity levels than do healthy individuals or whether implementing exercise therapy causes achieving sufficient daily physical activity levels for this population. We investigated steps/day as a measure of total physical activity levels in this group.

**Methods:**

Participants aged ≥20 years were identified from the Japanese National Health and Nutrition Examination Survey (2007-2019). Age-adjusted steps/day by health status were estimated using the analysis of covariance. The proportions of those meeting the physical activity recommendations (≥8,000 steps/day) among individuals with CCs who practiced exercise therapy were confirmed by density plots.

**Results:**

The data of 59,703 participants were analyzed. Age-adjusted steps/day in the multiple CC group (i.e., combinations of hypertension, diabetes, and dyslipidemia) were 480 steps/day fewer than those in the healthy group and 341 steps/day fewer than those in the single CC group. Furthermore, among the participants who engaged in exercise, the proportions of those not meeting physical activity guidelines were 51.2%, 56.7%, and 60.7% in the healthy, single CC, and multiple CC groups, respectively. Participants with diabetes (46.6%) showed the lowest proportion of those not meeting the physical activity guidelines among exercisers.

**Conclusions:**

Despite physical activity recommendations by clinical guidelines, steps/day in individuals with CCs were very low. More than half of individuals with CCs did not meet the physical activity guideline targets, even for those who reported engaging in exercise. This suggests that focusing only on exercise is not an appropriate strategy to increase total daily physical activity. Healthcare providers need to effectively promote physical activity in clinical settings, especially advocating for increasing not only exercise but also daily lifestyle physical activities.

## Introduction

Multiple chronic conditions (CCs), which refer to the presence of two or more chronic illnesses in one person ^(1), (2)^, cause an increased risk of mortality, loss of health-related quality of life, and functional decrease ^(3), (4), (5)^, in addition to high medical costs ^(6)^. Hypertension (HT) and dyslipidemia (DL) as cardiovascular conditions and diabetes mellitus (DM) as an endocrine condition often co-occur as multiple CCs ^(2)^. Clinical and public health guidelines recommend that individuals with CCs, including HT, DL, and DM, engage in exercise and increase physical activity (PA), as a modifiable lifestyle determinant of health ^(7), (8), (9)^.

Steps/day is a comprehensive measure of the total amount of daily PA. Accumulating more steps/day reduces all-cause mortality and cardiovascular disease rates, including in individuals with HT, DM, and DL ^(10), (11)^. The Japanese PA guidelines for individuals with CCs recommends ≥8,000 steps/day ^(9)^. Although individuals with CCs are recommended to increase PA ^(7), (8), (9)^, it remains unclear whether they reach these proposed targets. To date, normative reference data on steps/day for multiple CCs are lacking.

Furthermore, exercise is usually performed during leisure time, whereas total PA is performed in various domains, such as occupation, transportation, household chores, and leisure time ^(12)^. Even though some patients with CCs may perform ≥150 min/week of exercise and moderate-to-vigorous PA (i.e., achieving the recommendation for exercise therapy in clinical guidelines) ^(7)^ in the leisure-time domain, if they engage in less PA in the other domains ^(12)^, they may not accumulate sufficient total daily PA (i.e., ≥8,000 steps/day) ^(9)^. However, the number of individuals with CCs who exercise ≥150 min/week accumulate ≥8,000 steps/day is unclear.

In Japan, the National Health and Nutrition Survey (NHNS-J) provided data on steps and health status, including medication and blood sampling, from 2007 to 2019. Therefore, in this study, we aimed to describe the step-determined PA levels according to the presence of multiple CCs and medication status, stratified by sex and age categories (<65 and ≥65 years). Moreover, we examined the distribution of steps/day, especially among those who exercised ≥150 min/week, and presented the proportion of individuals taking 8,000 steps/day corresponding to the Japanese PA guidelines ^(9)^.

## Materials and Methods

The NHNS-J is a cross-sectional household interview and examination survey, including a pedometer survey. Detailed aspects of the survey have been described previously ^(13), (14)^. The survey comprised three parts: 1) physical examination, 2) nutritional assessment, and 3) lifestyle assessment. From 2007 to 2019, the same questionnaire on medical treatment was administered to the participants each year as part of their physical examination. Therefore, data from this period were used.

### Participants

The participant sampling design for the NHNS-J has been previously described ^(13), (14)^. Briefly, every year (except 2012 and 2016), the NHNS-J began by randomly selecting 300 census units from census enumeration areas previously selected as part of the Comprehensive Survey of Living Conditions of the People on Health and Welfare ^(15)^. Each census unit included approximately 20 households, and 5,000-6,000 households were sampled annually. Expanded surveys were conducted in 2012 and 2016 using a stratified single-stage cluster sample design to compare the NHNS-J results across 47 prefectures in Japan. For the 2012 and 2016 surveys, 23,750 and 24,187 households, respectively, were selected from 475 census units, comprising 10 areas in each prefecture (15 areas in Tokyo). Households with non-Japanese heads were excluded from the survey. Participants aged ≥20 years who participated in the pedometer survey and blood sampling during the physical examination were eligible. Participants who reported <500 or ≥50,000 steps/day or with missing data were excluded.

### Data collection

Individuals aged ≥20 years were invited to participate in the physical examination component of the NHNS-J. Participation in the blood sampling was voluntary. Data on blood pressure (BP) and blood parameters were obtained from the annual records of physical examinations in the NHNS-J. Medical doctors, public health nurses, and clinical laboratory technologists performed the physical examinations. Physical examinations included medical interviews and collecting information regarding medication status for HT, DL, DM, and exercise habits. Physical examinations were performed at designated community centers ^(16)^. BP was measured twice by the medical staff after the participants rested for at least 5 minutes in a sitting posture, according to the instructions of the NHNS-J. The mean of the two BP measurements was used for analysis. Non-fasting blood sampling was performed during the NHNS-J to encourage survey participation. In this study, six blood chemistry items, except for the 2012 and 2016 survey years, were available: total cholesterol (TC) (mg/dL), triglycerides (TGs) (mg/dL), high-density lipoprotein cholesterol (HDL-C) (mg/dL), low-density lipoprotein cholesterol (LDL-C) (mg/dL), glucose (mg/dL), and hemoglobin A1c (HbA1c) (%) ^(17)^. As for the expanded surveys conducted in 2012 and 2016, only four blood chemistry parameters were available: TC, HDL-C, LDL-C, and HbA1c. All blood samples were analyzed in a commercial testing laboratory ^(16)^. Exercise habits were assessed in terms of frequency (days per week) and duration per session ^(18)^. Information on exercise was measured through the following questions of the NHNS-J: (1) frequency of exercise: “How often do you exercise in a week?” (days/week); and (2) mean duration of exercise: “About how long do you exercise in each session?” (min/session). In this study, we calculated the total amount of exercise by multiplying the exercise frequency by the mean exercise duration (min/week). We categorized exercise duration (min/week) into three groups: 0, <150, and ≥150 min/week, based on the 2019 American College of Cardiology (ACC)/American Heart Association (AHA) guidelines on Primary Prevention of Cardiovascular Disease and the 2020 World Health Organization PA guidelines ^(7), (8)^.

### Classifications of the presence of CCs

We referred to established clinical practice guidelines to determine the presence or absence of CCs, such as HT, DM, and DL. HT was defined as the current use of antihypertensive medications, systolic BP ≥140 mmHg, or diastolic BP ≥90 mmHg ^(19)^. DM was defined as the use of antidiabetic medications, HbA1c of ≥6.5%, or casual plasma glucose levels of ≥200 mg/dL ^(20)^. DL was defined as the use of lipid-lowering medications, non-fasting/any time TG level of ≥175 mg/dL, LDL-C ≥140 mg/dL, HDL-C <40 mg/dL, or non-HDL-C ≥170 mg/dL ^(21)^. The non-HDL-C level was calculated using the following formula: non-HDL-C = TC − HDL-C. These were coded as binary variables representing the absence or presence of the condition. The HbA1c values from the Japan Diabetes Society were converted into National Glycohemoglobin Standardization Program equivalent values (%) ^(22)^.

### Pedometer survey

The same pedometer, AS-200 (Yamasa Co., Ltd., Tokyo, Japan), was used for the NHNS-J from 1995 to 2019. Yamasa is the generic Japanese name for Yamax--a commonly used research-grade pedometer ^(23)^. The pedometer survey was conducted on a single day between Monday and Saturday in November every year, selected by participants from the designated survey period (e.g., a weeklong period in November) and set by the survey office of each census unit. Previous studies have reported that for population-level surveillance, a single day is sufficient to generate stable group-level activity estimates ^(24), (25)^. Before each survey began, the survey staff distributed pedometers to each household (door-to-door) or at designated community centers near the surveyed areas. Participants were asked to wear the pedometer on their waist from the time they arose in the morning until they went to bed at night, only removing the device to engage in water-based activities. The participants recorded steps/day values in a survey log, which was returned on a scheduled physical examination day.

### Statistical analysis

Continuous variables are presented as means with standard deviations, and categorical variables as frequencies and proportions. We classified participants into three groups according to their health status: healthy, single CC, and multiple CCs. Age- and sex-adjusted steps/day for the healthy group and each CC type group were estimated using the analysis of covariance. Moreover, comparisons of steps/day between participants taking and not taking medication were performed to estimate the differences of patient education on PA-related lifestyle modification. This was because we assumed that participants taking medication for their CCs would attend a clinic as outpatients and receive exercise therapy from healthcare providers, whereas those not taking any medication would not necessarily attend clinics or receive exercise therapy. Therefore, the differences in steps/day between participants with and without medication could provide proxy information regarding the extent to which steps/day were promoted in clinical settings, including patient education for exercise therapy. In the subgroup analyses, we categorized the participants into four groups according to age and sex: men aged <65 years, women aged <65 years, men aged ≥65 years, and women aged ≥65 years. Furthermore, we compared the steps/day among participants who engaged in 0, <150, and ≥150 min/week of exercise, according to the ACC/AHA guideline recommendations (i.e., 150 min/week of exercise and PA) ^(7)^. Specifically, for the ≥150 min/week of exercise group, density plots of steps/day and proportion of ≥8,000 steps/day (corresponding to the Japanese guideline recommendations) ^(9)^ were graphically described using R version 4.1.2. All other statistical analyses were performed using IBM SPSS Statistics version 28 (SPSS Inc., Chicago, IL, USA) and R version 4.1.2 (R Core Team, Vienna, Austria). Statistical significance was set at p < 0.05.

### Ethical considerations

This study was a secondary analysis of anonymized data obtained by the Ministry of Health, Labour and Welfare (MHLW). The survey was conducted on the basis of the Health Promotion Law of Japan. The Ministry of Internal Affairs and Communications of Japan reviewed and approved the survey protocols, and informed consent was obtained from participants. The use of individual raw data from the NHNS-J from 2001 to 2019 was approved by the MHLW through official application procedures under Article 33 of the Japanese Statistics Act. Furthermore, the institutional review board of Tokyo Medical University waived the requirement for ethical review of this study owing to secondary data analysis.

## Results

Of 60,692 individuals who participated in the pedometer survey and blood sampling, 989 taking <500 or ≥50,000 steps/day were excluded. The data of 59,703 participants (mean age 58.6 ± 16.1 years, 40.8% were men) were analyzed (Table 1 and Supplementary Table 1). DM was less prevalent than HT and DL. In the total sample, 34.7% of participants took medication, and the proportion of participants taking medication was higher among those with multiple CCs than among those with a single CC (68.4% vs. 35.5%). Stratified by sex and age categories, the mean age values for men and women aged <65 and ≥65 years were 48.0 ± 12.2, 47.5 ± 11.7, 73.4 ± 6.0, and 73.3 ± 6.1, respectively (Supplementary Table 1). The rate of multiple CCs was higher in those older than 65 years and among men younger than 65 years than among their counterparts (Supplementary Table 1).

**Table 1.** Characteristics of Participants by Chronic Conditions.

					Types of CCs						
	Total sample	Healthy	Single CC	Multiple CCs	HT	DM	DL	HT and DM	HT and DL	DM and DL	HT and DM and DL
	(n = 59,703)	(n = 18,654)	(n = 22,404)	(n = 18,645)	(n = 10,566)	(n = 638)	(n = 11,200)	(n = 1,530)	(n = 13,010)	(n = 937)	(n = 3,168)
Sex, n (%)											
Men	24,379 (40.8)	5,606 (30.1)	9,883 (44.1)	8,890 (47.7)	5,341 (50.5)	382 (59.9)	4,160 (37.1)	1,000 (65.4)	5,723 (44)	477 (50.9)	1,690 (53.3)
Women	35,324 (59.2)	13,048 (69.9)	12,521 (55.9)	9,755 (52.3)	5,225 (49.5)	256 (40.1)	7,040 (62.9)	530 (34.6)	7,287 (56)	460 (49.1)	1,478 (46.7)
Age, y	58.6 (16.1)	47.4 (15.8)	61.0 (14.5)	66.8 (11.2)	66.3 (12.6)	67.1 (11.1)	55.7 (14.4)	70.3 (9.7)	66.2 (11.6)	64.5 (11.4)	68.3 (9.8)
Age category, n (%)											
<65 y	34,365 (57.6)	15,357 (82.3)	12,155 (54.3)	6,853 (36.8)	4,152 (39.3)	205 (32.1)	7,798 (69.6)	368 (24.1)	5,078 (39.0)	401 (42.8)	1,006 (31.8)
≥65 y	25,338 (42.4)	3,297 (17.7)	10,249 (45.7)	11,792 (63.2)	6,414 (60.7)	433 (67.9)	3,402 (30.4)	1,162 (75.9)	7,932 (61.0)	536 (57.2)	2,162 (68.2)
Systolic BP, mmHg	131 (19.1)	116.3 (11.8)	132.4 (17.8)	143.8 (16.3)	144.9 (15.5)	125 (10.1)	121.1 (11.1)	145.9 (16.7)	144.7 (15.8)	124.9 (9.7)	144.8 (16.3)
Diastolic BP, mmHg	78.8 (11.3)	72.9 (8.4)	79.8 (10.9)	83.6 (11.7)	84.9 (11.4)	73.8 (8.2)	75.4 (8.0)	80.7 (11.7)	85.1 (11.5)	75.1 (8.0)	81.6 (11.9)
TC, mg/dL	203.1 (35.3)	188.5 (25.6)	209.4 (35.7)	210.1 (38.6)	192.4 (25.5)	186.2 (26.0)	226.7 (35.9)	185.4 (25.7)	215.2 (38)	210.7 (41.1)	201.2 (39.2)
TG, mg/dL	136.6 (97.6)	85.3 (34.8)	138.8 (94.4)	181.1 (116)	98.2 (34.8)	96.2 (34.2)	173.7 (113.4)	105.8 (34.9)	182.4 (115.6)	194 (124.8)	203.5 (123.8)
HDL-C, mg/dL	61.8 (16.3)	67.7 (15.0)	61.6 (16.2)	56.2 (15.6)	64.6 (15.8)	60.6 (14.9)	58.9 (16.1)	59.9 (14.6)	56.8 (15.7)	54.0 (16)	52.4 (14.7)
LDL-C, mg/dL	118.4 (31.0)	104.9 (20.6)	124.3 (31.8)	125 (34.2)	107.2 (20.8)	105 (21.2)	141.4 (31.5)	103.3 (21.7)	129.3 (34.1)	125.8 (34.9)	117.6 (34.1)
Plasma glucose, mg/dL	105.3 (31.7)	95.3 (15.1)	101.8 (22.3)	118.5 (44.8)	102.6 (18.6)	152.3 (68.9)	98.5 (16.2)	151.1 (62.6)	104 (19.0)	154.9 (75.9)	153.5 (62.7)
HbA1c, %	5.7 (0.7)	5.4 (0.3)	5.6 (0.5)	6.2 (1.0)	5.6 (0.4)	7.1 (1.1)	5.6 (0.3)	7.1 (1.1)	5.7 (0.3)	7.4 (1.4)	7.2 (1.1)
non-HDL, mg/dL	141.3 (35.0)	120.8 (22.5)	147.8 (34.4)	154 (36.8)	127.8 (22.1)	125.6 (22.5)	167.8 (32.8)	125.5 (22.7)	158.4 (36.1)	156.7 (39.4)	148.8 (37.6)
Medication status											
Taking drugs, n (%)	20,706 (34.7)	0 (0.0)	7,944 (35.5)	12,762 (68.4)	5,536 (52.4)	371 (58.2)	2,037 (18.2)	1,242 (81.2)	8,287 (63.7)	607 (64.8)	2,626 (82.9)
Not taking drugs, n (%)	38,772 (64.9)	18,654 (100)	14,440 (64.5)	5,678 (30.5)	5,030 (47.6)	250 (39.2)	9,160 (81.8)	243 (15.9)	4,712 (36.2)	295 (31.5)	428 (13.5)
Missing, n (%)	225 (0.4)	0 (0.0)	20 (0.1)	205 (1.1)	0 (0.0)	17 (2.7)	3 (0.0)	45 (2.9)	11 (0.1)	35 (3.7)	114 (3.6)

Mean (SD) or n (%). BP: blood pressure; CC: chronic health condition (i.e.: HT, DM, or DL); DL: dyslipidemia; DM: diabetes mellitus; HbA1c: glycated hemoglobin A1c; HDL-C: high-density lipoprotein cholesterol; HT: hypertension; LDL-C: low-density lipoprotein cholesterol; non-HDL: non-high-density lipoprotein cholesterol; TC: total cholesterol; TG: blood triglyceride.

In participants with a single CC, steps/day were 139 steps/day fewer than in healthy participants (95% confidence interval: −219 to −59), whereas steps/day in participants with multiple CCs were 480 steps/day fewer (−569 to −390) (Table 2). The largest differences in steps/day were observed among male participants aged <65 years. Specifically, participants with multiple CCs took 1,324 (−1535 to −1113) fewer steps/day than the healthy group (Table 2). Steps/day in each CC are presented in Supplementary Table 2. Participants with DM had significantly more age- and sex-adjusted steps/day than did those with any other CC; however, there was no significant difference in steps/day between the healthy and DM groups.

**Table 2.** Comparisons of Age-Adjusted Steps/Day by Status of Chronic Conditions.

				Differences in steps/d		
	Healthy	With single CC	With multiple CCs	Healthy vs single CC	Single CC vs multiple CCs	Healthy vs multiple CCs
Total sample	6,801 (6,740-6,862)	6,662 (6,611-6,712)	6,321 (6,263-6,378)	-139 (−219 to −59)	-341 (−416 to −265)	-480 (−569 to −391)
Stratified by sex and age					
Men aged <65 y	8,292 (8,152-8,431)	7,703 (7,585-7,821)	6,968 (6,820-7,116)	−588 (−772 to −404)	−735 (−923 to −547)	−1,324 (−1,535 to −1,113)
Women aged <65 y	7,167 (7,096-7,238)	6,772 (6,685-6,860)	6,504 (6,372-6,636)	−395 (−512 to −277)	−268 (−421 to −116)	−663 (−820 to −506)
Men aged 65 y	6,454 (6,253-6,655)	6,164 (6,052-6,275)	5,806 (5,702-5,910)	−290 (−520 to −61)	−358 (−511 to −206)	−648 (−875 to −422)
Women aged 65 y	5,385 (5,240-5,530)	5,259 (5,176-5,343)	5,102 (5,024-5,180)	−126 (−293 to 42)	−158 (−272 to −43)	−283 (−449 to −118)

CC: chronic condition (i.e., HT, DM, or DL); DL: dyslipidemia; DM: diabetes mellitus; HT: hypertension.

**Figure 1.**
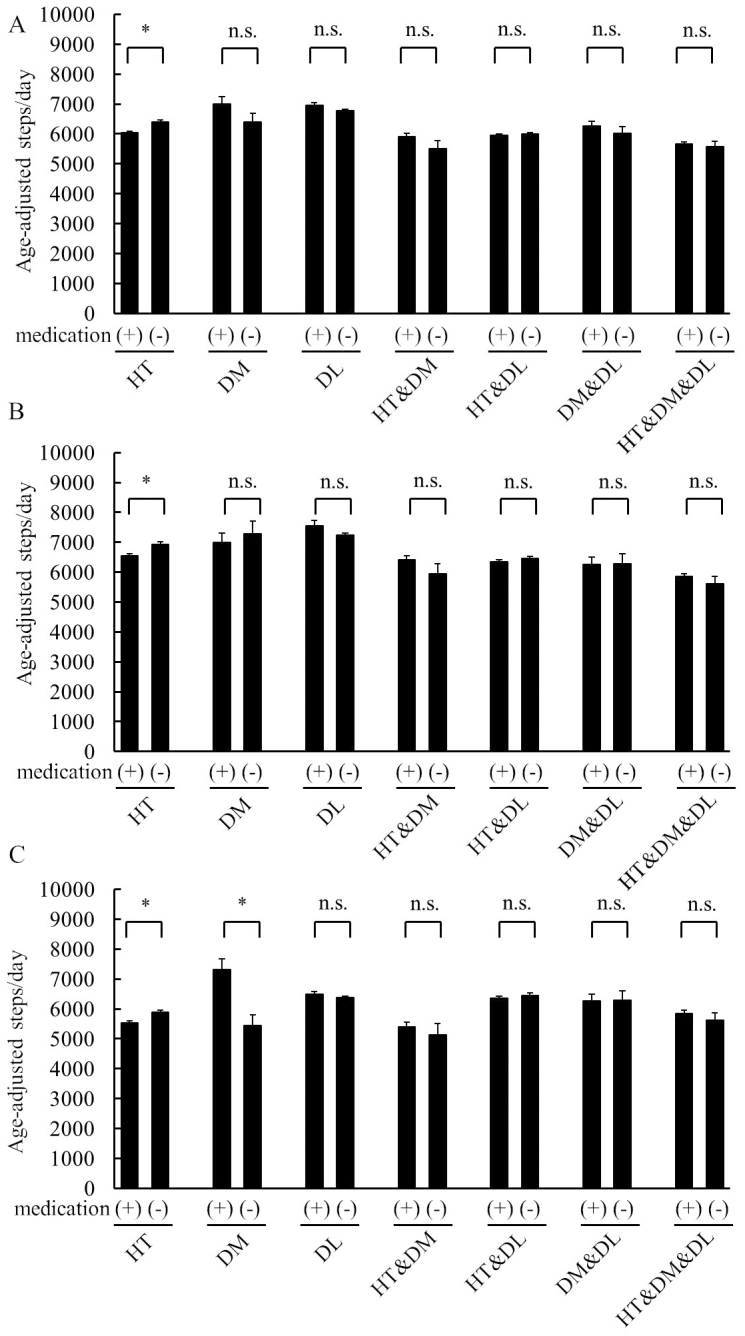
Comparisons of steps/day among participants with chronic conditions according to medication status (use/no use), stratified by sex and chronic condition. (A) Comparisons of steps/day by medication status in the entire sample. (B) Comparisons of steps/day by medication status in men. (C) Comparisons of steps/day by medication status in women. DL, dyslipidemia; DM, diabetes mellitus; HT, hypertension.

Comparisons of age-adjusted steps/day in those who took medication and those who did not are presented in Figure 1. In participants with HT, steps/day in those who used related medication were significantly lower than in those without any medication in both sexes. Conversely, among women, steps/day in participants with medication for DM were significantly higher than in those without medication. Among the remaining CCs, there were no significant differences in steps/day in both sexes.

Steps/day values by exercise category according to total exercise duration per week are presented in Supplementary Table 3. Steps/day gradually increased as participants exercised more in the healthy, single CC, and multiple CC groups. In the single CC group, no significant difference in steps/day was found between the 0 min/week and <150 min/week groups in men aged <65 and ≥65 years (Supplementary Table 3). In the multiple CC groups, participants who engaged in ≥150 min/week of exercise took 2,734 more steps/day than those who did not (Supplementary Table 3).

**Figure 2.**
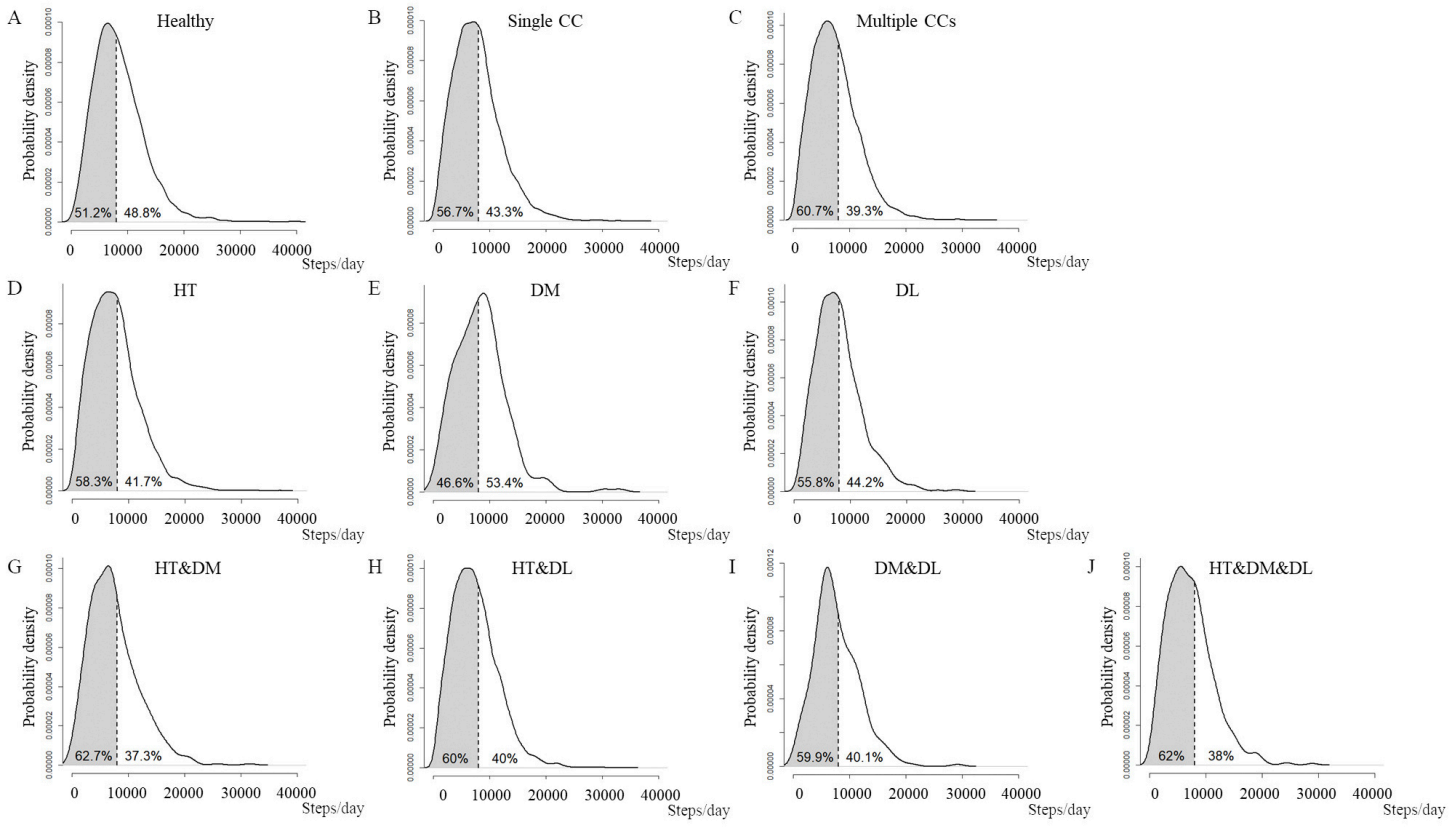
Density plot of steps/day in participants who engaged in ≥150 min/week of exercise, stratified by health status and CC type. The vertical dotted line indicates 8,000 steps/day. (A) Distribution of steps/day in the healthy group. (B) Distribution of steps/day in the single CC group. (C) Distribution of steps/day in the multiple CC group. (D) Distribution of steps/day in participants with HT. (E) Distribution of steps/day in participants with DM. (F) Distribution of steps/day in participants with DL. (G) Distribution of steps/day in participants with HT and DM. (H) Distribution of steps/day in participants with HT and DL. (I) Distribution of steps/day in participants with DM and DL. (J) Distribution of steps/day in participants with HT, DM, and DL. CC, chronic condition (HT, DM, or DL); DL, dyslipidemia; DM, diabetes mellitus; HT, hypertension.

In the participants who engaged in ≥150 min/week of exercise, the distributions of steps/day and proportion of taking fewer than 8,000 steps/day are presented in Figure 2. The proportions of participants who took fewer than 8,000 steps/day were 51.2%, 56.7%, and 60.7% in the healthy, single CC, and multiple CC groups, respectively, representing the proportions of participants who did not meet the Japanese PA guidelines. The proportion of participants who took fewer than 8,000 steps/day in the DM group (46.6%) was lower than that in other types of CCs (Figure 2). Similar results were observed in both sexes (Supplementary Figures 1 and 2). The median and interquartile range of steps/day in each health status among those who reported engaging in ≥150 min/week of exercise are listed in Supplementary Table 4.

## Discussion

We showed that 1) steps/day were lower in the multiple CC group than in the healthy and single CC groups, 2) steps/day between participants taking medication (i.e., visiting doctors) and those without medication were not significantly different across most multiple CCs, and 3) among those who reported engaging in ≥150 min/week of exercise, more than half of the participants did not achieve ≥8,000 steps/day ^(7), (8), (9)^. Our findings suggest that the current exercise prescription may not be effectively functioning for individuals with multiple CCs during outpatient care. Moreover, we revealed that self-reported exercise alone does not necessarily provide enough daily PA among individuals with CCs. Further PA promotion is warranted in clinical settings, especially in those with HT, DM, and DL.

Patient education based on behavioral theory may help promote PA in the clinical setting ^(26), (27)^. For example, goal setting and self-monitoring of behavior are commonly used approaches to behavior change ^(26)^. Steps/day is a simple yet comprehensive indicator of overall PA and can be easily measured by devices, including commercially available fitness trackers and smartphones ^(28)^. Information on steps/day can support goal setting by defining measurable targets (e.g., ≥8000 steps/day), which can be easily checked by either patients or clinicians. This structured approach can help patients and physicians understand personal health risks and choose suitable prevention strategies, based on the desired benefits, facilitating shared decision-making, which has been shown to help manage conditions such as HT, DM, and DL. ^(7)^ The availability of digital health technologies, which allow the monitoring of step counts and blood pressure values, among others, can help improve self-care and drive patient-centered outcomes by supporting treatment adherence and doctor-patient relationships ^(29), (30), (31)^.

In this study, in participants with DM, steps/day were highest in men aged ≥65 and higher for all women than for men aged <65 years or participants with other CCs. Moreover, women using medication for DM took more steps/day than those who were not. This suggests that PA promotion might be partially well-implemented for patients with DM, compared with other CCs. However, given there were fewer participants with DM than those with other CCs, the steps/day should be further evaluated in a larger sample. Conversely, participants taking medication for HT took fewer steps/day than those who did not. Because this study had a cross-sectional design, this may be partially explained by reverse causality. For instance, it is possible that participants with HT who were not taking medication engaged in more PA to thus help control their BP. Furthermore, steps/day were lower in women than in men among the healthy, single CC, and multiple CC groups. Our findings suggest that additional efforts are needed to promote PA among women, compared with men. Although this study suggests some sex-based differences in steps/day, PA levels achieved by individuals are often associated with personal, interpersonal, environmental, social, and policy-related factors ^(32), (33)^. Moreover, adherence to exercise habits differs by sociodemographic and psychosocial factors among participants with CCs ^(34), (35)^. Further studies accounting for sociodemographic factors are required to establish a suitable target for PA promotion among participants with CCs.

Because steps/day were lowest among participants with multiple CCs, exercise therapy is particularly warranted in this group. In participants with multiple CCs, those exercising ≥150 min/week took approximately 2,500 more steps/day than did those exercising 0 min/week. An increment of 1,000 steps/day was associated with a 9% lower risk of mortality (i.e., 5% and 15% mortality risk reduction in adults aged <70 and ≥70 years, respectively) ^(36)^. Moreover, a systematic review indicated that each 1,000-step increase was associated with a 5%-21% risk reduction in cardiovascular disease ^(37)^. The substantial effects of exercise in participants with multiple CCs should be expected.

In addition, recent studies proposed a phenomenon called “exercise resistance,” when insufficient baseline levels of PA render individuals resistant to the favorable improvements of cardiometabolic risk markers derived from exercise ^(38), (39)^. For instance, Burton and Coyle reported that 2 days of step reduction to 2,500-5,000 steps/day interfered with the beneficial effects of exercise on fat metabolism ^(38)^. Indeed, we observed that participants with multiple CCs who did not exercise took <5,000 steps/day. Clinical practitioners should also consider “exercise resistance” when prescribing exercise therapy ^(38), (39)^.

Furthermore, although the mean steps/day in participants with multiple CCs who reported engaging in ≥150 min/week of exercise exceeded ≥8,000 steps/day, corresponding to the recommendations of the Japanese PA guidelines ^(9)^, the results of the density plots showed that a substantial proportion of individuals with low steps/day (e.g., <8,000 steps/day) were included in the ≥150 min/week of exercise group. These findings suggest that self-reported engaging in ≥150 min/week of PA involving exercise alone did not accumulate enough PA overall in approximately half of the participants. Healthcare providers should advise their patients to increase their steps/day by engaging in not only exercise but also in more daily lifestyle activities (e.g., household chores, active commuting) ^(12)^, even if patients report ≥150 min/week of exercise. In accordance with the change of medical fees in Japan in 2024, clinicians are expected to provide comprehensive lifestyle-related disease management to outpatients, under the Lifestyle Disease Treatment Plan, including an exercise prescription ^(40)^, especially when managing HT, DM, and DL. A longitudinal study is needed to evaluate the effects of this medical insurance system revision on PA promotion aimed at preventing CCs.

### Limitations

This study has some limitations. First, this was a cross-sectional study, precluding inferences of causality. Fewer steps/day among people with multiple CCs could be both a cause and a consequence of poor health; however, this study provides a useful reference on the current PA status among patients with CCs using a nationally representative sample. Second, exercise habits were self-reported, thus potentially affected by recall and social desirability biases ^(41)^. Therefore, exercise habits might have been overestimated in this study. In addition, no data on the types of exercise were available. Pedometers would be removed if participants engaged in water-based activities (e.g., swimming), or full-contact sports (e.g., football, basketball, and martial arts), which would cause the total steps/day to be underestimated. Moreover, pedometers tend to underestimate steps/day achieved during cycling, which is a popular form of activity in Japan ^(42), (43)^. Third, given participation in the step survey and blood sampling were voluntary, steps/day might have been overestimated by selection (participation) bias ^(44)^. Fourth, we assumed that participants who did not take medication did not attend doctor visits, and that the differences in steps/day between medication status categories might reflect the effect of patient education on exercise therapy by doctors. However, some participants might have been visiting clinics for regular check-ups without receiving prescriptions; this would put them in a non-medication group, reducing the differences in steps/day between the groups. In contrast, participants receiving prescriptions might not have received PA counseling, the availability of which tends to vary depending on the diagnosed CC ^(45), (46)^, suggesting patient education regarding exercise therapy may differ among patients with CCs. Finally, CCs were not definitively diagnosed in clinical settings. In this study, we classified health status on the basis of clinical measurements made at one point in time; thus, the prevalence of CCs might have been overestimated. Moreover, in the expanded survey in 2012 and 2016, plasma glucose and TG were not evaluated, which could have caused diabetes and DL prevalence underestimations. Nevertheless, to maximize DL coverage, we used non-HDL-C.

### Conclusions

Despite PA recommendations by clinical and public health guidelines, individuals with CCs take approximately 500 steps/day fewer than do healthy individuals. Moreover, participants taking medication (i.e., visiting doctors) did not accumulate sufficient steps/day compared with those without medication, suggesting the need for improved patient education on exercise therapy in clinical settings. In addition, more than half of individuals with CCs did not meet the PA guidelines (≥8000 steps/day), even those who reported engaging in exercise, suggesting that focusing only on exercise is not an appropriate strategy to increase total daily PA. Healthcare providers need to promote PA in clinical settings, especially advocating for their patients to engage in not only exercise but also daily lifestyle PAs.

## Article Information

### Conflicts of Interest

None

### Author Contributions

Noritoshi Fukushima and Shigeru Inoue conceived the study. Noritoshi Fukushima and Shiho Amagasa performed data analysis. Misaki Takahashi supported data analysis. Noritoshi Fukushima drafted the initial manuscript. Shiho Amagasa, Misaki Takahashi, Hiroyuki Kikuchi, Rei Ono, and Shigeru Inoue interpreted the results and critically edited the manuscript. All authors read and approved the final manuscript.

### Ethical Statement/Informed Consent

This study was a secondary analysis of anonymized data obtained by the Ministry of Health, Labour and Welfare (MHLW). The survey was conducted on the basis of the Health Promotion Law of Japan. The Ministry of Internal Affairs and Communications of Japan reviewed and approved the survey protocols, and informed consent was obtained from the participants. The use of individual raw data from the NHNS-J from 2003 to 2019 was approved by the MHLW through official application procedures under Article 33 of the Japanese Statistics Act. Furthermore, the institutional review board waived the requirement for the ethical review of this study owing to its secondary use.

### Data Sharing

Data were obtained if the research was approved by the Ministry of Health, Labour and Welfare through official application procedures under Article 33 of the Japanese Statistics Act.

## Supplement

Supplementary Materials
