# Supplementary material for: Step-Determined Physical Activity among Individuals with Chronic Conditions: The 2007-2019 National Health and Nutrition Survey of Japan
Source: JMA J. 2025 Aug 22;8(4):1143–52. doi: 10.31662/jmaj.2025-0122 (PMC12598207; doi:10.31662/jmaj.2025-0122)
Supplement: Supplementary Materials [file 2433-3298-8-4-1143-s001.pdf]

Supplementary Table 1. Characteristics of participants, stratified by age category and sex

	<65 years		≥65 years	
	Men (n=13112)	Women (n=21253)	Men (n=11267)	Women (n=14071)
Age, years	48.0 (12.2)	47.5 (11.7)	73.4 (6.0)	73.3 (6.1)
Systolic BP, mmHg	130.8 (17.3)	121.7 (18.0)	140.0 (17.0)	138.0 (17.4)
Diastolic BP, mmHg	82.9 (11.5)	76.1 (11.2)	80.2 (10.9)	78.1 (10.5)
TC, mg/dL	202.1 (34.4)	206.2 (36.2)	191.6 (33.3)	208.5 (33.9)
TG, mg/dL	164.7 (126.5)	113.9 (81.2)	147.3 (92.3)	136.6 (84.6)
HDL-C, mg/dL	56.7 (15.3)	67.9 (15.6)	55.3 (15.2)	62.7 (15.4)
LDL-C, mg/dL	120.6 (31.3)	119.8 (31.8)	110.8 (29.1)	120.4 (30)
Plasma glucose, mg/dL	100.8 (31.7)	98.4 (23.5)	115.3 (39.9)	112.1 (31.6)
HbA1c, %	5.6 (0.7)	5.6 (0.6)	6.0 (0.8)	5.9 (0.7)
non-HDL, mg/dL	145.4 (36.2)	138.4 (36.2)	136.3 (32.5)	145.8 (32.9)
CC conditions, n (%)				
Healthy	4198 (32.0)	11159 (52.5)	1408 (12.5)	1889 (13.4)
With single CC	5293 (40.4)	6862 (32.3)	4590 (40.7)	5659 (40.2)
With multiple CCs	3621 (27.6)	3232 (15.2)	5269 (46.8)	6523 (46.4)
Types of CCs, n (%)				
HT	2155 (16.4)	1997 (9.4)	3186 (28.3)	3228 (22.9)
DM	102 (0.8)	103 (0.5)	280 (2.5)	153 (1.1)
DL	3036 (23.2)	4762 (22.4)	1124 (10)	2278 (16.2)
HT&DM	241 (1.8)	127 (0.6)	759 (6.7)	403 (2.9)
HT&DL	2576 (19.6)	2502 (11.8)	3147 (27.9)	4785 (34)
DM&DL	212 (1.6)	189 (0.9)	265 (2.4)	271 (1.9)
HT&DM&DL	592 (4.5)	414 (1.9)	1098 (9.7)	1064 (7.6)

Mean (SD) or number (%). BP; blood pressure, TC; total cholesterol, TG; blood triglyceride, HDL-C; high-density lipoprotein cholesterol, LDL-C; low-density lipoprotein cholesterol, HbA1c; glycated hemoglobin A1c, non-HDL; non-high-density lipoprotein cholesterol, HT; hypertension, DM; diabetes mellites, DL; dyslipidemia, CC; chronic condition (i.e., HT, DM, or DL).

Supplementary Table 2. Age-adjusted steps/day in participants in each chronic health condition.

	Healthy	HT	DM	DL	HT&DM	HT&DL	DM&DL	HT&DM&DL
Total sample	6806 (6744, 6868)	6592 (6517, 6666)	7220 (6924, 7516)	6694 (6623, 6765)	6426 (6233, 6619)	6357(6289, 6425)	6455 (6210, 6699)	6063 (5929, 6198)
Stratified by sex and age								
Men aged <65 years	8284 (8144, 8423)	7865 (7676, 8054)	7673 (6821, 8524)	7591 (7435, 7748)	7567 (7009, 8124)	7081(6909, 7253)	6624 (6034, 7215)	6399 (6041, 6757)
Women aged <65 years	7165 (7094, 7237)	6884 (6723, 7045)	7153 (6457, 7850)	6719 (6616, 6823)	6451 (5823, 7079)	6542(6394, 6690)	6580 (6066, 7095)	6282 (5932, 6633)
Men aged ≥65 years	6454 (6253, 6655)	6116 (5982, 6250)	6921 (6470, 7371)	6112 (5886, 6337)	6079 (5805, 6353)	5859(5724, 5993)	5887 (5423, 6350)	5445 (5217, 5672)
Women aged ≥65 years	5388 (5243, 5533)	5145 (5034, 5256)	6007 (5499, 6514)	5371 (5239, 5503)	5211 (4898, 5524)	5063(4972, 5154)	5453 (5071, 5835)	5142 (4949, 5334)

HT; hypertension, DM; diabetes mellites, DL; dyslipidemia,

Supplementary Table 3 age-adjusted mean steps/day (95% CI) among participants with chronic health conditions by engaging in exercise.

		0 min/week	<150 min/week	≥150 min/week	Differences in steps/day		
					0 vs 150 min/week	<150 vs ≥150min/week	0 vs ≥150min/week
Total sample							
	With no CC	6735 (6611,6859) [a,b]	7239 (7070,7408) [c]	8743 (8614,8872)	504 (300,708)	1504 (1295,1713)	2008 (1831,2186)
	With single CC	5712 (5602,5822) [a,b]	6133 (5994,6272) [c]	8006 (7912,8101)	420 (244,597)	1873 (1705,2042)	2294 (2147,2440)
	With multiple CCs	4787 (4674,4899) [a,b]	5524 (5388,5660)[c]	7521 (7430,7612)	737 (560,914)	1997 (1833,2161)	2734 (2589,2879)
Men aged <65 years							
	With no CC	8091 (7796,8386)[b]	8112 (7696,8528) [c]	9412 (9116,9708)	21 (-489,531)	1300 (789,1811)	1321 (903,1739)
	With single CC	7241 (6985,7497) [b]	7449 (7089,7809) [c]	9071 (8813,9330)	207 (-234,649)	1623 (1179,2066)	1830 (1466,2195)
	With multiple CCs	6033 (5751,6314) [a,b]	6733 (6320,7146) [c]	8642 (8362,8922)	700 (201,1199)	1909 (1409,2409)	2609 (2210,3008)
Women aged <65 years							
	With no CC	6610 (6473,6747) [a,b]	7197 (6987,7407) [c]	8783 (8600,8966)	587 (336,838)	1586 (1309,1863)	2173 (1943,2403)
	With single CC	6200 (6035,6365) [a,b]	6641 (6413,6869) [c]	8474 (8287,8662)	441 (158,723)	1833 (1539,2127)	2274 (2022,2526)
	With multiple CCs	5897 (5642,6151) [a,b]	6517 (6198,6837) [c]	8441 (8181,8702)	621 (212,1030)	1924 (1512,2335)	2545 (2179,2910)
Men aged ≥65 years							
	With no CC	5164 (4691,5637) [a,b]	6047 (5492,6601) [c]	7753 (7452,8055)	882 (153,1612)	1707 (1075,2338)	2589 (2028,3150)
	With single CC	5020 (4762,5278) [b]	5306 (4957,5656) [c]	7620 (7452,7787)	286 (-148,721)	2313 (1925,2701)	2599 (2292,2907)
	With multiple CCs	4457 (4233,4681) [a,b]	5048 (4762,5335) [c]	7433 (7284,7582)	591 (227,955)	2385 (2062,2708)	2976 (2707,3245)
Women aged ≥65 years							
	With no CC	4612 (4252,4971) [a,b]	5481 (5128,5833) [c]	7074 (6817,7330)	869 (365,1373)	1593 (1157,2029)	2462 (2021,2904)
	With single CC	4590 (4384,4797) [a,b]	4983 (4777,5189) [c]	6697 (6548,6846)	392 (101,684)	1714 (1460,1968)	2106 (1851,2362)
	With multiple CCs	4113 (3944,4282) [a,b]	4909 (4731,5086) [c]	6556 (6423,6690)	796 (551,1041)	1648 (1426,1870)	2444 (2228,2659)

HT; hypertension, DM; diabetes mellites, DL; dyslipidemia, CHC; chronic health condition (i.e., HT, DM or DL), LTPA; leisure-time physical activity. Values were adjusted for age and sex in total sample and the remains were adjusted for age by ANCOVA.

[a] p<0.05 between 0 min/week and <150 min/week of exercise,

[b] p<0.05 between 0 min/week and 150 min/week of exercise,

[c] p<0.05 between <150 min/week and 150 min/week of exercise.

Supplementary Table 4. The 25th, 50th, and 75th percentiles of steps/day for each health status among participants who reported engaging ≥150 min/week by sex.

	Total sample			Men			Women		
	25%tile	50%tile	75%tile	25%tile	50%tile	75%tile	25%tile	50%tile	75%tile
Healthy	5302	7850	10924	5537	8038	11255	5205	7620	10695
Single CC	4786	7291	10096	4948	7545	10580	4617	7018	9634
Multiple CCs	4421	6910	9733	4554	7116	10202	4263	6651	9205
HT	4434	7130	9968	4731	7532	10489	4016	6512	9226
DM	5329	8213	10692	5075	8149	10635	5786	8435	10732
DL	5044	7384	10160	5092	7530	10673	5018	7281	9864
HT&DM	4250	6787	9928	4426	6884	10040	3937	6502	9270
HT&DL	4471	6958	9768	4654	7273	10332	4235	6615	9224
DM&DL	5038	7004	10202	5516	7441	10673	4957	6572	9580
HT&DM&DL	4228	6651	9508	4230	6532	9687	4216	6859	8961

CC, chronic condition (i.e., HT, DM, or DL); HT, hypertension; DM, diabetes mellitus; DL, dyslipidemia.

Supplementary Fig. 1 Density plot of steps/day in male participants who engaged in ≥150 min/week of exercise, stratified by health status and CC type.

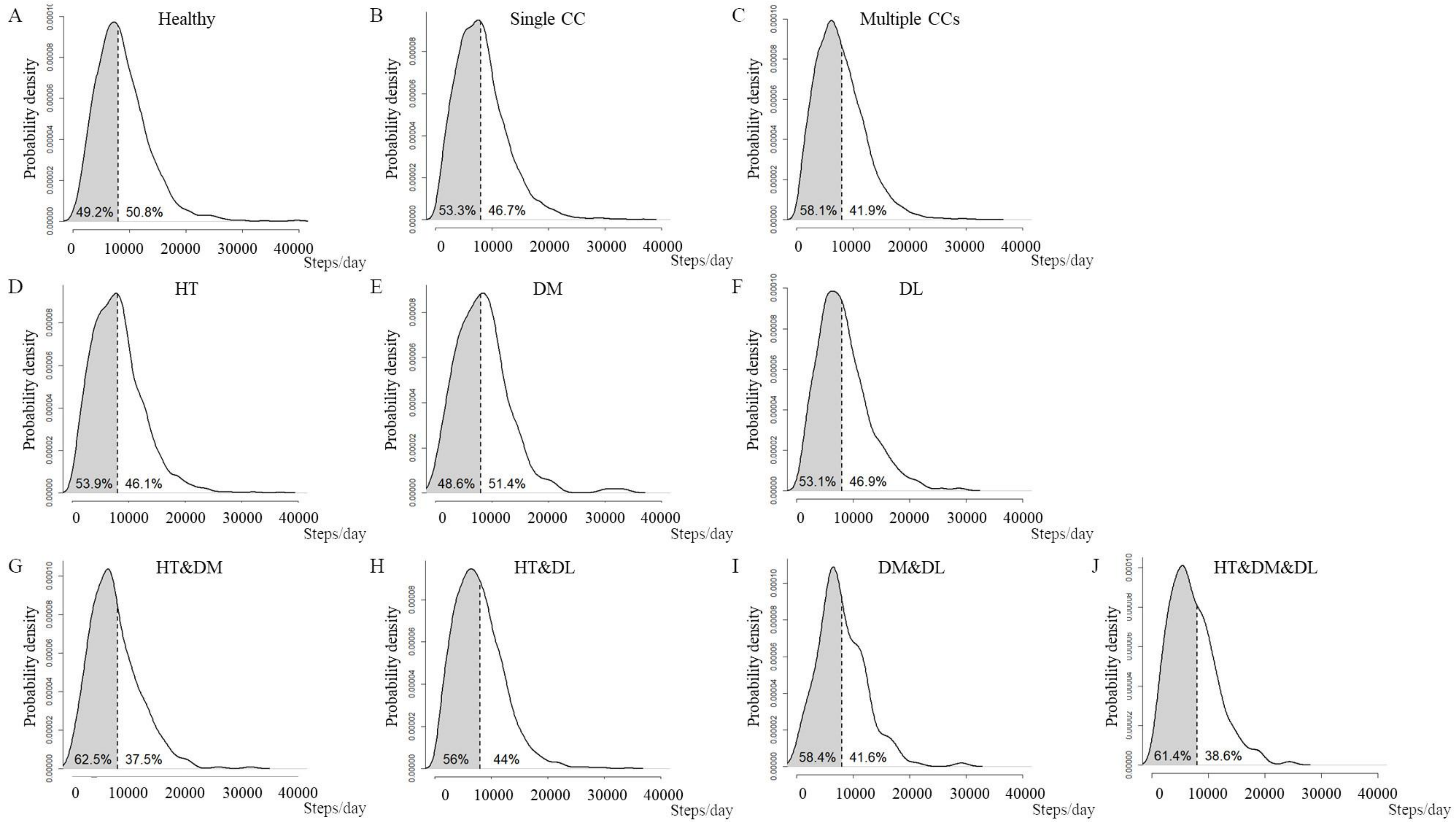

Density plot of steps/day in male participants who engaged in ≥150 min/week of exercise, stratified by health status and CC type. The vertical dotted line indicates 8,000 steps/day. (A) Distribution of steps/day in the healthy group. (B) Distribution of steps/day in the single CC group. (C) Distribution of steps/day in the multiple CC group. (D) Distribution of steps/day in participants with HT. (E) Distribution of steps/day in participants with DM. (F) Distribution of steps/day in participants with DL. (G) Distribution of steps/day in participants with HT and DM. (H) Distribution of steps/day in participants with HT and DL. (I) Distribution of steps/day in participants with DM and DL. (J) Distribution of steps/day in participants with HT, DM, and DL. HT, hypertension; DM, diabetes mellitus; DL, dyslipidemia; CC, chronic condition (HT, DM, or DL).

Supplementary Fig. 2 Density plot of steps/day in female participants who engaged in ≥150 min/week of exercise, stratified by health status and CC type.

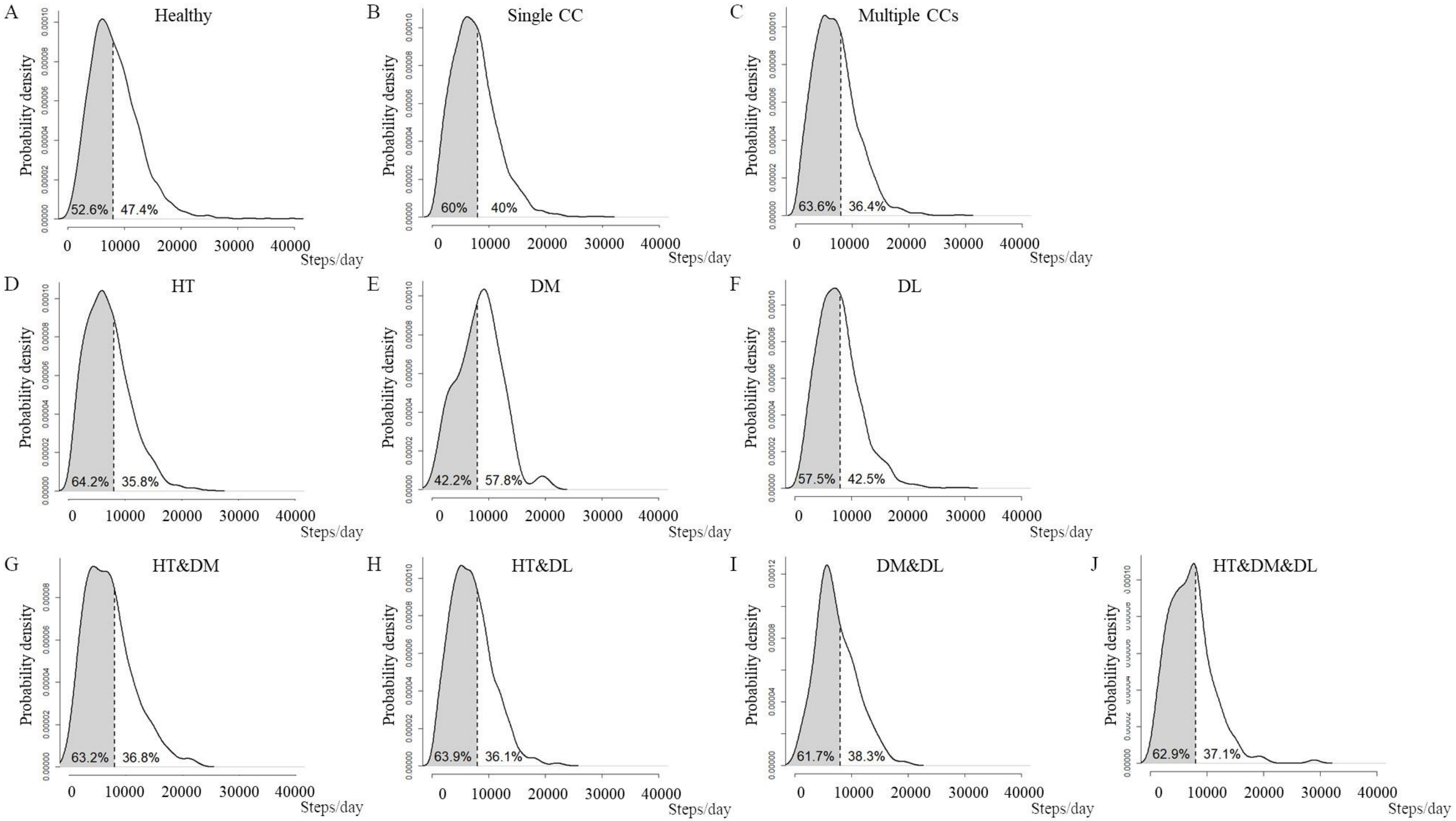

Density plot of steps/day in female participants who engaged in ≥150 min/week of exercise, stratified by health status and CC type. The vertical dotted line indicates 8,000 steps/day. (A) Distribution of steps/day in the healthy group. (B) Distribution of steps/day in the single CC group. (C) Distribution of steps/day in the multiple CC group. (D) Distribution of steps/day in participants with HT. (E) Distribution of steps/day in participants with DM. (F) Distribution of steps/day in participants with DL. (G) Distribution of steps/day in participants with HT and DM. (H) Distribution of steps/day in participants with HT and DL. (I) Distribution of steps/day in participants with DM and DL. (J) Distribution of steps/day in participants with HT, DM, and DL. HT, hypertension; DM, diabetes mellitus; DL, dyslipidemia; CC, chronic condition (HT, DM, or DL).
